# A Comparison of the Efficacy and Cost of Different Venous Leg Ulcer Dressings: A Retrospective Cohort Study

**DOI:** 10.1155/2015/187531

**Published:** 2015-04-14

**Authors:** Syed M. Asim Hussain

**Affiliations:** North Cumbria University Hospitals NHS Trust, Cumberland Infirmary, Newtown Road, Carlisle, Cumbria CA2 7HY, UK

## Abstract

*Objective*. To compare the efficacy and cost-effectiveness of simple nonadherent dressings with other more expensive dressing types in the treatment of venous leg ulcers. *Study Design*. Retrospective cohort study. *Location*. The leg ulcer clinic at the University Hospital of South Manchester. *Subjects and Methods*. The healing rates of twelve leg ulcer patients treated with simple nonadherent dressings (e.g., NA Ultra) were compared with an equal number of patients treated with modern dressings to determine differences in healing rates and cost. *Main Outcome Measures*. Rate of healing as determined by reduction in ulcer area over a specified period of time and total cost of dressing per patient. *Results*. Simple nonadherent dressings had a mean healing rate of 0.353 cm^2^/week (standard deviation ± 0.319) compared with a mean of 0.415 cm^2^/week (standard deviation ± 0.383) for more expensive dressings. This resulted in a one-tailed *p* value of 0.251 and a two-tailed *p* value of 0.508. Multiple regression analysis gave a significance *F* of 0.8134. *Conclusion*. The results indicate that the difference in healing rate between simple and modern dressings is not statistically significant. Therefore, the cost of dressing type should be an important factor influencing dressing selection.

## 1. Introduction

**Figure 1:**
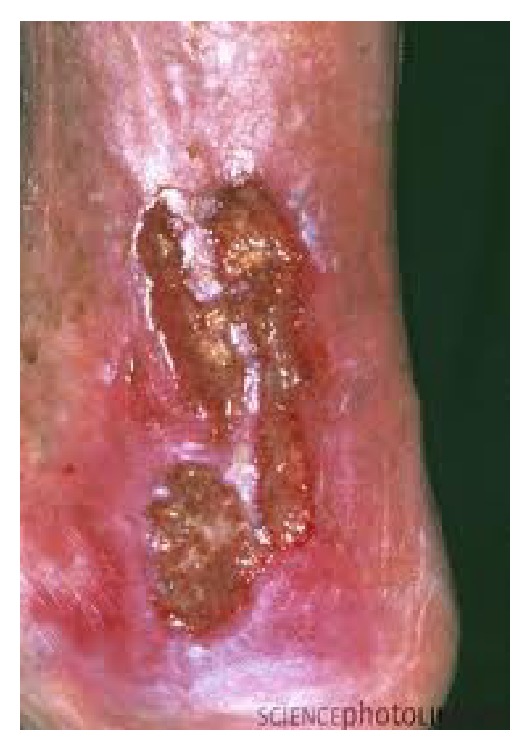
Venous leg ulcers are usually located in the “gaiter” region of the leg as shown in the image above. They have certain characteristics: (i) ruddy coloured base, (ii) large area, (iii) shallow depth, (iv) irregular wound margins, (v) moderate to heavy exudates, (vi) pitting or nonpitting oedema, (vii) granulation tissue, (viii) warm skin temperature, and (ix) pulses present with normal capillary refill of less than 2 seconds [13]. Some of these can affect the rate of wound healing. They can be associated with other signs of venous hypertension such as varicosities, haemosiderin pigmentation, atrophy blanche, and varicose eczema [13, 14].

A venous ulcer is a break in the continuity of the skin resulting from venous hypertension [1] (Figure 1). This is the most common cause of leg ulceration in the United Kingdom with a prevalence of 1.5–3 per 1000, increasing to 20 per 1000 in patients over 80 years old [1]. About 48% of venous leg ulcers recur after 5 years [2]. This presents significant disease burden to the United Kingdom National Health Service with an estimated cost of *£*300 million/year [3]. Therefore, both therapeutic efficacy and cost-effectiveness should be taken into account when creating evidence-based management plans.

*Aetiology of Venous Ulceration*. Incompetent valves in either the superficial venous system or the perforator veins result in chronic venous hypertension followed by ulceration. There is dispute concerning the final mechanism of ulceration. The original “fibrin cuff” hypothesis proposed that venous hypertension causes fibrinogen to leak out and build up around the vessel preventing oxygen and nutrients from reaching the cells [4]. This was followed by the “leucocyte trapping” theory [5], which attributes tissue damage to trapped white cells releasing proteolytic enzymes and oxygen free radicals. The latest theory suggests chronic inflammation due to repetitive ischaemia-reperfusion as the primary mechanism of cellular damage and wound formation [6]. Regardless of the exact pathogenesis, treatment involves reversing the venous hypertension causing the ulcer. 

*Venous Mapping in Ulcer Diagnosis*. Venous disease of the lower legs can be classified using clinical severity, aetiology, anatomy, and pathophysiology (CEAF classification system). The role of Doppler ultrasound in evaluating venous disease according to the CEAF classification has demonstrated very low intraobserver variability and a high sensitivity and specificity [7]. Patients with an open venous ulcer are already classified as having the most clinically severe form of venous disease (C6 classification). This form of venous disease is significantly associated with both reflux and thrombosis.

*Treatment of Venous Ulcers*. Conservative management of venous ulcers involves elevating the legs and wearing graduated compression stockings to reduce venous stasis and oedema [8]. Current evidence suggests that multilayered compression is more effective than single layer compression [8]. A 40 mmHg compression at the ankle has been found to be most effective [9]. Four layered bandages increase the chance of healing by 30% compared to short stretch bandages [10].

Dressings for venous ulcers are chosen on the basis of ability to absorb fluid and odour, adhesiveness, antibacterial and haemostatic properties, potential to cause sensitivity reactions, ease of handling, tendency to shed fibres, and interval required between dressing changes [11]. Modern dressings have been developed with these characteristics in mind. Semiocclusive dressings like hydrogels enhance autolytic debridement. Silver, iodine, or honey based dressings have increased antimicrobial activity and hydrocolloids increase the moisture content of the wound [12]. Whether these dressings actually do promote faster wound healing by the above mechanisms is up for debate.

A trial of this nature has not been done at University Hospital of South Manchester leg ulcer clinic. A retrospective comparison was made to assess differences in efficacy and cost-effectiveness between simple nonadhesive Ultra dressings (e.g., NA Ultra) and modern dressings.

## 2. Methods

### 2.1. Null Hypothesis

There is no significant difference in leg ulcer healing rates when comparing simple nonadherent Ultra dressings with modern dressings such as Inadine, Iodoflex, Medihoney, Aquacel Ag, and Atrauman Ag.

### 2.2. Alternative Hypothesis

Modern dressings significantly improve leg ulcer healing rates compared to simple nonadherent Ultra dressings.

### 2.3. Inclusion Criteria

Venous leg ulcer patients showing a measurable change in area over time were included.

### 2.4. Exclusion Criteria

Patients with an Ankle Brachial Pressure Index (ABPI) <0.8 or a history of deep vein thrombosis, diabetes mellitus, inability to move, or mental health problems were excluded.

Patient medical records were collected and divided into two groups: those treated with “simple nonadherent Ultra dressings” and those treated with “modern” dressings. Records that did not satisfy the inclusion criteria were eliminated leaving a total of 24 patients. Twelve were treated with simple nonadherent Ultra dressings and the rest were treated with an array of different dressings including silver based dressings such as Atrauman Ag and Aquacel Ag, iodine based dressings such as Iodoflex and Inadine, and the honey based Medihoney dressing.

According to medical records all venous ulcer patients underwent a complete history and examination. The wound was assessed for slough, granulation, epithelialisation, and level of exudate. The surrounding skin was assessed for dryness, eczema, and haemosiderin pigmentation. The calf and ankle circumference were measured and appropriate compression therapy started. Dressings were changed weekly and the ulcers reviewed. The ulcer area was calculated every week by tracing the ulcer on graph paper and counting the squares. Pictures were taken to keep a visual record of the healing process and the percentage of granulation tissue was noted.

The time taken to complete healing, defined as 100% epithelialisation of the wound, was calculated in weeks according to the record of weekly reviews. For some patients data was lacking and the time to complete healing could not be determined. In these patients the time period between two measured ulcer areas was taken and the healing rate was calculated as change in area/time taken for that change in cm^2^/week. The average healing rate and standard deviation were calculated for both groups. The unpaired Student's *t*-test was applied to determine significance assuming an equal variance between both groups.

The cost of dressings for treatment was also measured by multiplying the total area of dressing used for the treatment period by the cost of dressing per unit area. The cost of dressings is subsidised by the NHS supply line. Access to these prices was denied. The NHS supply line prices were estimated to be 50% of the market price based on knowledge of the price of Inadine in both the market and NHS supply line. The market price was standardised as the price at http://dressingsonline.com/. The area of dressing used was calculated by multiplying the initial ulcer area with the number of dressings changed until the ulcer healed. The average cost of dressings during the treatment period and the standard deviation were calculated for both groups. The unpaired Student's *t*-test was applied to determine significance assuming an equal variance in both groups. Cohen's *d* and the effect size *r* were also calculated to evaluate the strength of difference. The average cost of dressings per patient was used to calculate the annual cost of dressings of both types. Yearly savings of using simple nonadhesive dressings were then calculated.

## 3. Results

There were 6 males and 6 females in the simple nonadherent Ultra group compared to 2 males and 10 females in the other group. The average age was 68.67 years for the simple nonadherent group compared with an average age of 71.5 years in the other group. Eighteen of the 24 patients had completely healed ulcers. Seven were from the simple nonadherent Ultra dressing group and 11 were from the group containing other dressing types. The data available was not sufficient to determine if the remaining 6 patients' ulcers completely healed.

The healing rate was usually less than 1 cm^2^ per week and more than 0.1 cm^2^ per week independent of the type of dressing used. There are anomalies in both groups where ulcer healing rate exceeds 1 cm^2^ per week. In the simple nonadhesive Ultra dressing group there is an anomalous decreased value for the healing rate equal to 0.02 cm^2^ per week. It is possible that these anomalies resulted from factors other than the type of dressing used such as the duration of the ulcer, the type of compression therapy, or patient compliance.

The mean healing rate for simple nonadherent Ultra dressings is 0.353 cm^2^ per week with a standard deviation of 0.3185 (Table 1). This is less than the mean healing rate for other dressing types calculated to be 0.415 cm^2^ per week with a standard deviation of 0.383 (Table 2). The unpaired Student's *t*-test was applied to determine the significance of this difference with 22 degrees of freedom. This resulted in a one-tailed *p* value of 0.336 and a two-tailed *p* value of 0.672, indicating that the difference in ulcer healing between the two groups is not statistically significant (Table 3).

A multiple regression analysis was also performed to validate the data (Table 4). This gave a significance *F* value of 0.8134 with the coefficient of determination *R* ^2^ = 0.0058. This supports the findings of Student's *t*-test suggesting no significant difference in venous ulcer healing rates.

The cost of dressings was also estimated. Total costs of simple nonadherent Ultra dressings range from *£*0.009 to *£*3.82, while modern dressings range from less than *£*0.2 to more than *£*8, increasing with initial ulcer area and duration of treatment. The mean cost of dressing using nonadherent Ultra is *£*0.702 with a standard deviation of 1.08 (Table 1) compared with a mean cost of *£*4.78 and a standard deviation of 4.816 using other dressings (Table 2). This gives a one-tailed *p* value of 0.0045 on application of Student's *t*-test (Table 3). Cohen's *d* is 1.17 and the effect size *r* is calculated to be 0.5 which is conventionally classed as “medium.” An estimated *£*100000 per year can be saved by treating 25000 patients with simple nonadherent dressings instead of other more expensive dressings (Table 5).

## 4. Discussion

Many prospective randomised controlled trials have compared modern dressings with simple nonadhesive dressings, for example, NA Ultra. A meta-analysis published in Wound Repair and Regeneration evaluated 31 studies comparing polyurethane, activated charcoal, alginates, hydrocolloids, and collagen dressings with conventional dressings and found no significant differences in wound healing [16]. Another meta-analysis published in BMJ evaluated 42 randomised trials to determine the efficacy of hydrocolloids, hydrogels, alginates, and foams [17]. Most of the trials were limited by small sample size. There was no significant difference in healing when hydrocolloids and simple dressings were compared, but there was not enough data to draw strong conclusions for other dressing types. Another systematic review found that semiocclusive dressings such as foam, film, cellulose, alginate, and hyaluronic acid derived dressings were not more effective than simple low adherent dressings in improving healing rates in venous ulcer patients [18]. It also found no statistical differences in the proportion of ulcers healed with silver based dressings compared to dressings not containing silver. This study supplements these trials by providing a comparison of ulcer healing rates with different dressings in the Greater Manchester patient population.

Ulcer healing is a complex and dynamic process that includes clotting, inflammation, granulation tissue formation, epithelialisation, neovascularisation, collagen synthesis, and wound contraction [19]. The ideal environment for wound healing is adequately oxygenated, warm, and moist and is free from infection or necrotic tissue [19].

The results indicate that leg ulcer healing rates do not significantly vary depending on the type of dressing used while significant savings in cost can be made by the increased use of simple nonadherent Ultra dressings.

In the current economic climate there is an urgent need to make the best use of available resources. This requires healthcare practitioners to identify where cuts can be made without affecting patient care. The use of modern dressings is one such area as all available evidence suggests that the cheaper simple nonadhesive dressings work just as well [20]. This is supported by the VULCAN randomised controlled trial which found that silver impregnated dressings have an incremental cost of *£*97.85 compared to simple dressings without significantly improving healing rates [21].

Cost of dressing shows wide variation. However, estimated yearly savings of more than *£*200000 can be made if 50000 leg ulcer patients per year are treated with NA Ultra dressings instead of modern expensive dressings (Table 5). Small changes in the price of dressings can cause large differences in the total cost as it is dependent on duration of treatment and ulcer size as well.

Of note, the ulcer area is generally greater in the modern dressings group resulting in greater mean cost. The significant cost difference cannot be ascribed to this alone. In this study it is assumed that the same area of dressing is used at each follow-up visit. In reality, the area of dressing used is likely to be less each week as some ulcer healing would have taken place. This is countered by the assumption that all of the dressing area was used to cover the ulcer without any waste. This likely overcompensates for the initial assumption resulting in an underestimate of the dressing costs and yearly savings. This was done for the sake of simplicity as consistent application of the same formula would not affect the comparison in costs between both groups.

*Limitations of the Study*. Most major factors affecting wound healing (Table 6) were controlled through the exclusion criteria. However, important confounding factors that were not controlled include BMI, ulcer duration, type of compression therapy, patient compliance, smoking and alcohol intake, and wound infection. Nonetheless, the two groups are still comparable given that these factors are likely evenly distributed amongst both groups.

Since this is a retrospective study, ulcer area calculation and management were not biased by the motives of the research decreasing the likelihood of confounding due to different treatments. However, the retrospective nature of the study does open itself to bias in data selection and misclassification. To minimize this, selection of patient records was randomized by asking an individual not involved in the research to hand over the patient records once the exclusion criteria had been satisfied.

The use of different comparators for the “modern dressings” group may also make the comparison unfair. It is possible that one of the many modern dressings is significantly better than simple dressings while another is significantly worse making the cumulative effect negligible. However, given that all the modern dressings are antimicrobial in nature we can reasonably treat them as a single group and compare them collectively. The lack of patients treated with a particular type of dressing makes individual comparison difficult even though such a comparison would be ideal.

The small sample size (*n* = 24) is a limitation shared by previous studies on this subject. This is difficult to overcome unless a similar study is repeated with a much larger patient population covering many different clinical sites. Most venous ulcer patients have multiple comorbid conditions and thus need to be excluded from studies to avoid confounding factors. Our study included 24 patients with an open venous ulcer. This is the most clinically severe form of venous disease (C6 classification) which would give the largest scope for improvement. A large sample size is necessary if effect sizes are small. However, if effect size is indeed small then it would not be cost-effective to use very expensive dressings for potentially little benefit.

Based on these results it is not altogether clear if a large scale randomised controlled trial to determine efficacy and cost-effectiveness of dressings is warranted. All studies undertaken so far have been consistent in concluding that healing rates do not depend on type of dressing. However, most of these have suffered from methodological limitations. There is also a tendency to intuitively think antimicrobial dressings or moisture enhancing dressings would improve healing rates given that infection and dryness impair healing. This is coupled with anecdotal evidence and individual claims that modern dressings work and simple dressings do not. A cost-benefit analysis is suggested for a large scale prospective study comparing specific dressings in uncomplicated venous ulcer patients.

## 5. Conclusion

There is no significant difference in healing rates for different types of dressings. The cost of dressing should be the primary factor influencing dressing selection unless the patient prefers a particular dressing. Patients should be informed that there is no conclusive evidence that modern dressings provide superior wound healing.

## Figures and Tables

**Table 1:** Results for simple nonadherent Ultra dressings. The mean healing rate was 0.353 cm^2^/week with a standard deviation of 0.3185 and the mean cost of dressings was 0.702 GBP with a standard deviation of 1.08. The standard error was 0.092.

Age	Gender	Initial ulcer area [cm^2^]	Final ulcer area [cm^2^]	Change in ulcer area [cm^2^]	Duration of change in area [weeks]	Rate of healing [cm^2^/week]	Dressing type	Cost of dressing per patient [GBP]
66	F	6	0	6	26	0.23	Ultra	1.21
79	M	2	0	2	8	0.25	Ultra	0.12
69	M	1.4	0	1.4	17	0.08	Ultra	0.19
80	M	28.8	7.9	20.9	17	1.23	Ultra	3.82
82	F	3.6	0.3	3.3	17	0.19	Ultra	0.48
81	F	7.6	3.2	4.4	23	0.19	Ultra	1.36
50	F	1.2	0	1.2	2	0.6	Ultra	0.02
70	F	3.2	0	3.2	8	0.4	Ultra	0.20
64	M	3.2	0	3.2	12	0.27	Ultra	0.30
53	M	6.46	2.3	4.16	13	0.32	Ultra	0.66
82	F	0.15	0	0.15	8	0.02	Ultra	0.009
48	M	2.0	0.2	1.8	4	0.46	Ultra	0.06

**Table 2:** Results for different types of modern dressings. The mean healing rate was 0.415 cm^2^/week with a standard deviation of 0.383 and the mean cost of dressings was 4.78 GBP with a standard deviation of 4.816. The standard error was 0.111.

Age	Gender	Initial ulcer area [cm^2^]	Final ulcer area [cm^2^]	Change in ulcer area [cm^2^]	Duration of change [weeks]	Rate of healing [cm^2^/weeks]	Dressing type	Estimated cost of dressing per patient [GBP]
88	M	2.5	0	2.5	3	0.83	Atrauman Ag	0.15
79	M	3.9	0	3.9	16	0.24	Inadine	0.52
58	F	0.8	0	0.8	8	0.1	Atrauman Ag	0.13
63	F	1.2	0	1.2	9	0.13	Urgotul	1.2
78	F	5	0	5	22	0.23	Medihoney	6.6
51	F	8.8	0	8.8	18	0.49	Iodoflex	9.2
79	F	13.5	0	13.5	17	0.79	Medihoney	13.8
84	F	5.85	0	5.85	17	0.34	Aquacel Ag	9.54
80	F	11.5	6	11.5	4	1.34	Iodoflex	2.67
59	F	4	0	4	26	0.15	Aquacel Ag	9.98
88	F	2.24	0	2.24	13	0.17	Medihoney, Actifoam	3.33
51	F	2.21	0	2.21	13	0.17	Inadine	0.24

**Table 3:** Cost is significantly different between simple dressings and modern dressings. However the healing rate between the two is not significantly different.

	Simple nonadherent Ultra dressing (*n* = 12)	Other types of dressing (*n* − 12)	Degrees of freedom (2*n* − 2)	One-tailed *p* value	Two-tailed *p* value	Cohen's *d*
Mean healing rate Standard deviation of healing rate	0.353 0.3185	0.415 0.383	22	0.336	0.672	0.176

Mean cost dressing/*£* Standard deviation of cost	0.702 1.08	4.78 4.816	22	0.0045	0.009	1.17

**(a):** 

ANOVA					
	df	SS	MS	*F*	Significance *F*
Regression	1	0.006517	0.0065169	0.058734699	0.813405139
Residual	10	1.10955	0.110955		
Total	**11**	**1.116067**			

**(b):** 

Regression statistics	
Multiple *R*	0.076414
*R* square	0.005839
Adjusted *R* square	−0.09358
Standard error	0.333099
Observations	12

**Table 5:** The amount of money that can be saved depending on the number of ulcer patients treated per year.

Number of ulcer patients treated/year	*n* = 10000	*n* = 50000	*n* = 75000	*n* = 100000
Mean yearly cost of simple NA Ultra dressing/*£*	7020	35100	52650	70200

Mean yearly cost of other dressings	47800	239000	358500	478000

Yearly saving using NA Ultra	40780	203900	305850	407800

**Table 6:** Some of the factors that affect wound healing [15].

Systemic factors which impair wound healing	Local factors which impair wound healing
Inadequate oxygenation Increased BMI Increased age Sex hormones: aged males have been shown to have delayed healing Psychosocial stress causing delayed healing Comorbidities, heart disease, TIA, and diabetes Glucocorticoids, immunosuppressants, and NSAIDs Chemotherapy and anticoagulants Alcohol consumption impairing wound healing Smoking impairing wound healing Deficiency of carbohydrates, proteins, vitamins A, B, and C, iron, zinc, copper, and magnesium	Infection and pus Necrotic tissue and foreign bodies Dry wound Mechanical stress on wound Cold environmental temperature Local oxygenation: this is decreased in peripheral vascular disease, previous radiation, inflammation, and diabetes mellitus
